# Data-driven modeling of cellular stimulation, signaling and output response in RAW 264.7 cells

**DOI:** 10.1186/1750-2187-3-11

**Published:** 2008-05-22

**Authors:** Yang Wu, Gary L Johnson, Shawn M Gomez

**Affiliations:** 1 Joint Department of Biomedical Engineering, University of North Carolina School of Medicine, Chapel Hill, North Carolina, USA; 2 Department of Pharmacology, University of North Carolina School of Medicine, Chapel Hill, North Carolina, USA; 3 Lineberger Comprehensive Cancer Center, University of North Carolina School of Medicine, Chapel Hill, North Carolina, USA; 4 Center for Comparative Medicine and Translational Research, College of Veterinary Medicine, North Carolina State University, Raleigh, North Carolina, USA

## Abstract

**Background:**

Understanding the relative importance of signaling pathway components which regulate a specific cellular response is a major focus of current efforts in biology. This interest, along with the inherit complexity of these systems, is driving the development of approaches capable of providing both quantitative predictions as well as guiding the design of future experiments. Of particular interest is the establishment of methods for the analysis of cellular-level input-output signaling relationships that have been characterized over time.

**Results:**

Work by the Alliance for Cellular Signaling (AfCS) has provided an extensive profile of ligand-induced changes in protein phosphorylation state and cytokine output response in macrophage-like RAW 264.7 cells. Using model averaging with partial least squares (PLS) or principal components regression (PCR), we compared multivariate models quantitatively predicting cytokine release and identifying key regulatory components of the underlying signaling pathways. We paid particular attention to the effect of metrics extracted from the experimentally derived signaling time courses so as to determine whether the inclusion of such temporal information improved model predictions. Results indicate that we were able to determine the key biological predictors responsible for generating a specific cytokine response, with model *R*^2 ^values ranging from 0.48 to 0.93. Furthermore, for this data set, the use of time metrics was found to be of mixed value, with increased and/or more appropriate sampling likely being required to improve predictive performance.

**Conclusion:**

The use of multivariate approaches and model averaging provides a valuable predictive framework for quantitative studies of these complex biological processes. Results of this work also point to several issues for consideration in the design of similar large-scale interrogations.

## Introduction

A continuing challenge in biology today is the need to integrate large quantities of experimental data into quantitative and testable descriptions of system behavior. Such a challenge is particularly relevant at the cellular level, where recent technological advancements have made the generation of large-scale and comprehensive data sets feasible. Given such data, the opportunity arises to greatly improve our understanding of the overall dynamics of cellular behavior and its relevance to cellular dysfunction. Due to their size and complexity, it is generally recognized that the data generated through large-scale interrogations are largely uninterpretable without the use of computational methods for data reduction, analysis and modeling. As a result, a number of methods have been adopted from fields such as engineering, computer science and statistics, which are particularly well suited for dealing with such systems-scale biological data [1,2]. For example, recent work by Sachs and colleagues [3] used Bayesian networks to predict causal network relationships between proteins involved in T cell signaling, while multivariate approaches such as partial least squares (PLS) regression have been used for identifying and modeling key components of cytokine-induced apoptosis [4-6].

Recent work by the Alliance for Cellular Signaling (AfCS) has led to the generation of an extensive, openly accessible, profile of the system-wide response of macrophage-like RAW 264.7 cells to over 200 input stimuli. These stimuli were applied to cells either alone or simultaneously as a paired combination, with the resulting changes in cytokine output responses quantified over time. In addition to the cytokine outputs, the phosphorylation states of 21 signaling proteins were also characterized over time. Overall, such data presents a large-scale picture of cell system dynamics that is still relatively rare in the literature. Herein, we model the input/output response of RAW 264.7 cells based on the studies performed by the AfCS. Due to possible advantages of the method (described below), we use partial least squares regression for modeling input/output responses, and compare these results with identical analyses using principal components regression (PCR). We were particularly interested in the temporal aspects of the data as recent work by Janes and colleagues [4,5] has shown that the use of parameters derived from temporal response curves, such as time derivatives, peak value, and area under the curve (AUC), were typically more informative than time-averaged data. This question is especially relevant here as the AfCS data is composed of cytokine response curves and phosphorylation state measurements consisting of only 4 time points (typically sampled at 0, 2, 3, and 4 hours for cytokines and 1, 3, 10 and 30 minutes for phosphorylation). These are relatively sparsely sampled time curves and it is not obvious if this amount of sampling is sufficient to generate reliable results or relative improvements when compared to time-averaged data.

Here we show that for this data, the predictive capability of PLS and PCR were generally equivalent. However, there was a significant benefit of PLS over PCR in the significant reduction in the number of variables that must be used within the models to accurately describe variation within the data. In addition, the ability to generate variable importance in projection (VIP) scores with PLS provides the ability to readily determine important variables (e.g. specific signaling molecules) that drive cellular output response. The generation and interpretation of these VIP scores is much simpler than methods developed to adapt PCR to this task [7]. We found that the use of time-derived metrics was of marginal utility here, most likely due to the low sampling of the signaling response curves. Finally, in the course of this analysis we identified several issues in the design of the experiments that generated this data. We suggest possible changes for future studies that can improve the quality of analyses and interpretation of the experimental results in such large-scale interrogations.

## Results

### Data overview

As discussed in greater detail in Materials and Methods, the AfCS data was derived from RAW 264.7 macrophage-like cells and consisted of phosphorylation state time courses for 21 signaling proteins and the resulting release of seven cytokines including G-CSF, IL-1*α*, IL-6, IL-10, MIP-1*α*, RANTES and TNF*α*. In the process of our analysis of the data, we became cognizant of several important issues. In total there were 253 stimulating conditions, 22 single and an additional 231 applied to cells as a pair (see Materials and Methods). As both the phosphorylation state of intracellular proteins and the cytokine output response were measured, the stimulatory inputs were performed twice; once to generate the phosphorylation data and once to generate the cytokine data. Unfortunately, for the majority of cases the input stimuli were not matched across experiments – i.e. the concentration(s) of stimulants was not the same across both the phosphorylation and cytokine response experiments. Differences were significant (e.g. commonly 3–5 fold). Of the initial 253 sets of experiments, only 55 had appropriately matched input conditions. While we decided that 55 conditions were sufficient for the work addressed here, this finding severely limited the greater utility of the larger data set.

### PLS and PCR

A method common in the field of chemometrics, PLS is an extension to the multiple linear regression model and thus related to other methods including principal components regression [8]. The main goal of these methods is to describe a linear model, *Y *= *X B *+ *E*, where *Y *is a *n *object by *m *variable response/output matrix, *X *is a *n *by *p *variable predictor matrix, *B *is a *p *by *m *regression coefficient matrix and *E *is a noise term. Here, *X *and *Y *are the independent and dependent blocks respectively. In this work, *Y *includes measurements of the cytokine output response, while the *X *block consists of measurements of signaling protein phosphorylation state.

Both PLS and PCR produce factor scores *T *(*T *= *XW*; *W *is a weight matrix), which are linear combinations of the original predictor variables and are thus uncorrelated with each other. These components also encapsulate correlated observed variables within a single new constructed component (i.e. the so called "latent variable" in PLS and "principal component" in PCR) and thus help to reduce issues common to high-dimensional data sets. Regression is performed on these components, thus *Y *= *TQ *+ *E*, where *Q *is a matrix of regression coefficients (loadings) for *T*. Once the coefficients are calculated, this model is equivalent to the original and can be used in prediction. An important aspect is that PCR uses components that maximally describe variation in *X *alone. PLS differs from PCR in that it tries to find components that are the best compromise between both fitting *X *and predicting *Y *(the independent and dependent blocks respectively). Thus PLS tries to find factors that both capture variance and achieve correlation with both predictor and predicted variables. While highly data-dependent, in general one would expect PLS to outperform PCR if the data have a large amount of variance that is nonlinear and/or unrelated to the dependent variables. In addition, the fact that PLS uses both independent and dependent blocks X and Y, one would generally expect PLS to perform better than PCR with the input/output data collected in the AfCS study. As described below, we found that this is not necessarily the case for this data.

### Modeling of cytokine output response

We first wanted to look at what effect using temporal information had on prediction accuracy for both PLS and PCR. In this case, we extracted time-dependent signaling metrics from the time curves that describe the phosphorylation state of the 21 intracellular signaling proteins (see Table 1). These 11 metrics provide a potentially greater capability to identify more physically relevant variables in the signaling process. For instance, it is likely that time-related properties of a signaling protein, such as its peak activity level, the rate of change in activation, or the total amount of activity is/are the critical factors in deciding whether or not a given cytokine response is triggered. With 11 metrics extracted from the phosphorylation curves of each of the 21 signaling proteins, there can be a maximum of 231 variables represented within a PLS or PCR model. For comparison purposes we also consider models developed using time-averaged signaling measurements. Note that while the inclusion of time-dependent metrics would seem to be the most appropriate methodology, insufficient experimental sampling of these time curves can lead to models with low predictive accuracy, in which case models derived from time-averaged data may be more appropriate. We used 10-fold cross-validation to develop the PLS and PCR models (see Materials and Methods).

**Figure 1:**
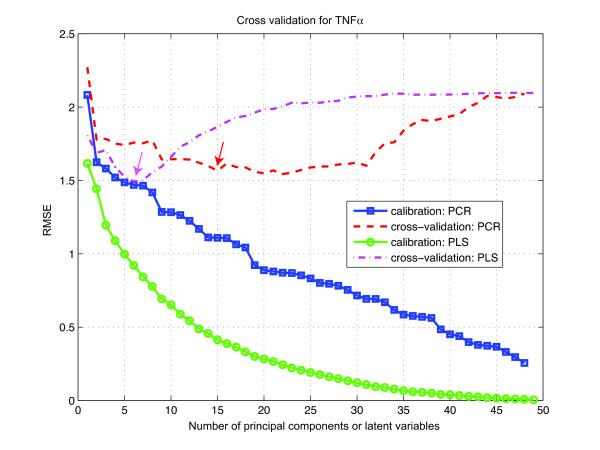
**Root-mean-square errors of calibration and cross-validation of TNF*α *response with both PCR and PLS analysis**. Time-dependent signaling metrics were used. Arrows indicate minimum errors and hence the number of components used for the regression models.

Briefly, the data was split into ten equally sized contiguous blocks. All but one of the blocks were used to train the model (calibration stage), with the resulting model then being used to predict the withheld block (test stage). To choose an optimized number of latent variables (LVs) or principal components (PCs), we examined the root-mean-square error (RMSE) between the measured and the predicted responses with increasing numbers of LVs or PCs for each cytokine. As LVs or PCs, which describe large amounts of systematic variance (i.e. variables of predictive value), are added to the model, the cross-validation RMSE (RMSECV) should decrease. On the other hand, when LVs or PCs describing only small noise variance are added (i.e. variables that are largely noise), the RMSECV should increase. For example, when time-dependent signaling metrics were used to predict the output response of TNF*α *secretion, the calibrated RMSE decreased monotonically, while the RMSECV was minimized with just 6 LVs for the PLS model and with 22 PCs for the PCR model (Figure 1). However, as can be seen in the figure, the decrease of RMSECV in the PCR model after 15 PCs was relatively modest, which suggests that we can use 15 PCs rather than 22 and still achieve good prediction accuracy. This emphasizes a significant benefit of multivariate approaches such as PLS and PCR which is their ability to accurately model system behavior with a reduced set of critical variables. These critical variables then represent the most important factors driving system behavior and output response.

**Table 1:** Metrics extracted from protein phosphorylation state time courses.

Metric class	Metrics generated
Temporal measurements	1 min
	3 min
	10 min
	30 min

Instantaneous derivatives	1 min
	3 min
	10 min
	30 min

Summary metrics	area under the curve (AUC)
	Maximum signal
	Mean signal

#### Prediction accuracy

After selecting the optimized number of LVs or PCs for each model of cytokine output response, we examined the squared Pearson correlation coefficient, *R*^2^, between measured cytokine outputs and the cross-validated predictions (Table 2). Overall, high correlation could be achieved in all predictions. Specifically, high *R*^2 ^values were found for G-CSF and TNF*α *(*R*^2 ^ranging from 0.72 to 0.93), while moderate *R*^2 ^values were found for IL-6, IL-10, MIP-1*α *and RANTES (*R*^2 ^ranging from 0.48 to 0.65) using either regression method with time-dependent signaling metrics. PLS or PCR predictions with time-dependent signaling metrics were weakest for the IL-1*α *response with *R*^2 ^values ranging from 0.49 to 0.51. On the other hand, PLS or PCR predictions with time averages were much better for this particular cytokine response (0.83 and 0.85 respectively).

**Table 2:** Prediction accuracy as measured by squared Pearson correlation coefficient *R*^2^

	G-CSF	IL-1*α*	IL-6	IL-10	MIP-1*α*	RANTES	TNF*α*
PLS (time-derived metrics)	0.93 (5)	0.49 (1)	0.63 (5)	0.62 (4)	0.49 (6)	0.65 (2)	0.75 (6)
PCR (time-derived metrics)	0.92 (2)	0.51 (2)	0.64 (21)	0.62 (16)	0.48 (10)	0.64 (2)	0.72 (15)
PLS (time-averaged data)	0.88 (5)	0.83 (6)	0.58 (6)	0.64 (5)	0.62 (5)	0.73 (5)	0.83 (5)
PCR (time-averaged data)	0.89 (10)	0.85 (10)	0.58 (13)	0.68 (10)	0.59 (9)	0.77 (9)	0.84 (13)

Number of principal components (for PCR) or latent variables (for PLS) are listed in parentheses.

The results of Table 2 show that, despite having measurements of output response that could be utilized by the PLS model, PCR was found to marginally outperform PLS in 5 of 7 cytokine output responses. Furthermore, the results of using time-dependent signaling metrics were generally poor with prediction accuracy improving for only 2 of 7 cytokine outputs (G-CSF and IL-6). In the remaining 5 outputs, time-averaged models had significantly better predictive power. When compared to the PCR models, PLS regression achieved a much smaller model dimension. While the order of PCR models ranged from 2 to 21, the order of PLS models ranged from 1 to 6 while still achieving a similar RMSECV level for all 7 cytokines (Table 2). Thus in general, the PLS model requires a smaller number of variables than PCR to achieve nearly the same level of prediction accuracy, producing the simplest or most "minimal" models as a result.

### Vital signaling metric selection

**Figure 2:**
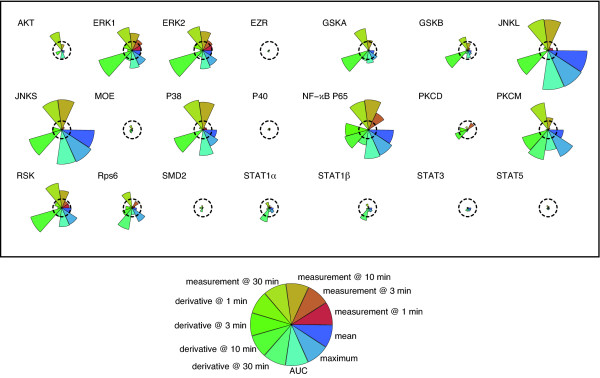
**Squared weighted VIP profile for RANTES**. Ten PLS models were generated through 10-fold cross validation and then a weighted VIP score was computed as described in Materials and Methods to select the important signaling metrics. A segment plot was produced for each protein, with the radial length of each segment indicating the magnitude of the squared weighted VIP score for individual metrics. VIP scores greater than 1 (dashed circle) are classified as significant metrics. For example, here we see that the mean, maximum and AUC for JNKL/S activity are the most informative metrics for RANTES, while proteins such as EZR do not have predictive value under the conditions studied. see that the mean, maximum and AUC for JNKL/S activity are the most informative metrics for RANTES, while proteins such as EZR do not have predictive value under the conditions studied.

**Figure 3:**
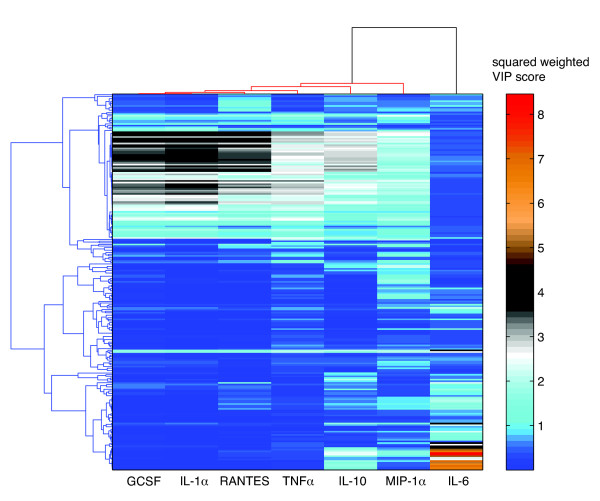
**Clustering of squared weighted VIP profiles for all seven cytokines**. Two-way average linkage clustering was performed using uncentered Pearson's correlation distances.

A benefit of the PLS approach is the ability to readily determine the important/highly predictive variables within a model. We do this by calculating the weighted VIP score for each cytokine (see Materials and Methods). An example of this is shown in Figure 2, which shows the squared weighted VIP score profile for RANTES. This profile shows the ranking of all 231 variables in the RANTES PLS model, with the two most influential variables being the maximum value and area under the curve for the JNK phosphorylation state time course [profiles for the remaining 6 cytokines are provided in Additional file 1]. Note that the determination of highly predictive variables is a very straightforward process in the PLS methodology when compared to other approaches with PCR (e.g. [7]). Figure 3 shows the global squared weighted VIP profile patterns for all cytokine responses. We note that, in practice, identified VIPs may span a significant range with regard to their information content and predictive capacity. As a result, only some smaller fraction of the highest-ranked VIP scores is kept for use in the model as well as for further analysis. The top 10% and top 20% signaling metrics for each cytokine are shown in Table 3 and discussed further below.

**Table 3:** Top 10% and 20% most significant time-dependent signaling metrics as identified via PLS

Cytokine	Top 10% metrics	Top 20% metrics (not including those in top 10%)
G-CSF	JNK lg: AUC, maximum	JNK lg: mean, @ 30 min, derivative @ 10 min, @ 10 min
	JNK sh: AUC	JNK sh: derivative @ 10 min, maximum, mean, @ 30 min, @ 10 min
		P38: @ 30 min, @ 10 min
		ERK1: derivative @ 10 min
		ERK2: derivative @ 10 min
		RSK: derivative @ 10 min, @ 30 min
		NF-*κ*B p65: @ 10 min, AUC, @ 30 min, maximum
		PKCM: derivative @ 30 min

IL-1*α*	JNK lg: maximum	JNK lg: AUC, @ 30 min, mean, derivative @ 10 min, @ 10 min
	JNK sh: AUC	JNK sh: maximum, mean, derivative @ 10 min, @ 30 min, @ 10 min
		ERK1: derivative @ 10 min
		ERK2: derivative @ 10 min
		P38: @ 30 min
		RSK: derivative @ 10 min
		PKCM: derivative @ 30 min
		NF-*κ*B p65: @ 10 min, AUC, maximum, @ 30 min

IL-6	STAT3: mean, AUC, derivative @ 3 min, @ 3 min, maximum, @ 1 min, derivative @ 1 min, @ 10 min	STAT3: @ 30 min
		STAT1*α*: derivative @ 10 min
		STAT1*β*: derivative @ 10 min

IL-10	RSK: derivative @ 10 min	
	ERK2: derivative @ 10 min	
	ERK1: derivative @ 10 min	
	JNK sh: derivative @ 10 min, @ 30 min, AUC, @ 10 min	JNKsh: maximum, mean
	JNK lg: @ 30 min, @ 10 min, derivative @ 10 min	JNK lg: AUC, maximum, mean
	P38: derivative @ 10 min	P38: @ 30 min
	NF-*κ*B p65: AUC	NF-*κ*B p65: maximum, @ 30 min, @ 10 min, mean
		GSK3A: derivative @ 10 min

MIP-1*α*	JNK lg: mean, maximum, AUC	
	NF-*κ*B p65: @ 30 min, derivative @ 30 min	NF-*κ*B p65: @ 10 min
	JNK sh: maximum, AUC	JNK sh: mean, derivative @ 10 min
	STAT5: 1 min, derivative @ 1 min	
		ERK2: derivative @ 10 min
		ERK1: derivative @ 10 min
		PKCM: maximum, derivative @ 30 min
		P38 @ 30 min, maximum, @ 10 min
		STAT1*α*: derivative @ 30 min

RANTES	JNK lg: maximum, AUC, mean	JNK lg: derivative @ 10 min, @ 30 min, @ 10 min
	JNK sh: maximum, AUC, derivative @ 10 min	JNK sh: mean, @ 10 min, @ 30 min
	ERK2: derivative @ 10 min	
	ERK1: derivative @ 10 min	
		RSK: derivative @ 10 min
		PKCM: derivative @ 30 min, maximum
		P38: derivative @ 10 min, @ 30 min
		NF-*κ*B p65: @ 10 min

TNF*α*	NF-*κ*B p65: @ 30 min, @ 10 min	
	JNK lg: mean, maximum, AUC	
		PKCM: derivative @ 30 min, @ 30 min, maximum
		P38: @ 30 min, @ 10 min, AUC
		JNK sh: maximum, AUC, mean
		RSK: @ 30 min
		ERK1: @ 30 min
		Rps6: derivative @ 30 min
		ERK2: @ 30 min

### Redundant encoding of signaling metrics

To examine the redundancy in the signaling information contained within the original 231-metric model, we generated PLS models using only the reduced set of metrics with VIP scores greater than 1. We found that for each cytokine, a PLS model containing from 49 up to 97 most informative signaling metrics was as predictive as the complete one that used all 231 metrics (Table 4) implying that there is significant redundancy in the information carried by each metric. Similarly, we also assessed the quality of prediction as a function of the number of vital metrics used in the regression. Model uncertainty was estimated by randomly shuffling samples 500 times. The averaged *R*^2 ^increases as the number of the vital metrics included in the regression also increases (data not shown). As shown in Table 5, we found that for each cytokine, a PLS model containing from 1 to 46 of the most informative metrics (as determined by VIP score) can achieve an averaged *R*^2 ^greater than 85% of the maximum averaged *R*^2^. Using fewer than those most informative metrics, however, could not predict the cytokines with the desired accuracy.

**Table 4:** Prediction results of PLS regression using all vital signaling metrics

	G-CSF	IL-1*α*	IL-6	IL-10	MIP-1*α*	RANTES	TNF*α*
number of vital metrics	74	70	49	85	97	76	78
*R*^2^	0.90	0.49	0.72	0.66	0.54	0.69	0.76

For each cytokine, a PLS regression model using from 49 to 97 most informative (vital) signaling metrics was as predictive as the complete model that used all 231 metrics.

**Table 5:** Prediction results of PLS regression using top vital signaling metrics.

	G-CSF	IL-1*α*	IL-6	IL-10	MIP-1*α*	RANTES	TNF*α*
Numb. of metrics	1	1	14	34	46	1	38
*R*^2^	88.0 ± 0.27%	51.4 ± 0.55%	64.2 ± 2.41%	56.6 ± 2.61%	49.4 ± 2.42%	75.0 ± 0.60%	66.3 ± 1.84%

For each cytokine, a PLS regression model using from 1 to 46 of the most informative signaling metrics can achieve an average *R*^2 ^greater than 85% of the maximum averaged *R*^2^. Values are means ± standard deviation. Numb. of metrics = Number of most informative metrics used in the regression.

### Identification of primary signaling network components

A benefit of dimension reduction and regression models such as PLS is that they provide the capability to identify key modulators behind a specific signaling response. For example, model predictions for G-CSF were the most accurate of all cytokines, with *R*^2 ^values ranging as high as 0.93 for the PLS model using time-derived metrics (Table 2). G-CSF, which is secreted by T cells, macrophages, endothelial cells, and bone marrow stroma, acts on bone marrow progenitor cells, inducing the differentiation of myeloid precursors into mature granulocytes as well as having other immune function [9,10]. For this system, we found that the key modulator of the G-CSF response (i.e. within the top 10% most informative metrics) was the overall total activity level of JNK (considered here to be represented by the AUC and maximum properties, see Table 3). G-CSF is induced by nuclear factor *κ*B (NF-*κ*B) and activation protein-1 (AP1), and the CCAAT enhancer-binding protein, C/EBP*β*, (NF-IL6 in humans) has also been shown to bind to the G-CSF promoter site [11-13]. C/EBP*β *in turn is activated via a JNK-dependent mechanism [14]. A second example is TNF*α*. Tumor necrosis factor is a primary mediator of the acute inflammatory response, with macrophages and T cells being the main biological sources [9,11]. Physiologically, TNF*α *stimulates the recruitment of leukocytes to sites of infection and/or inflammation and also promotes their activation. The most potent stimulus for eliciting a TNF response from macrophages is LPS, which is a stimulus condition that was an appropriately matched set within the AfCS data, and thus was used within these models. Binding of TNF*α *to appropriate receptors leads to recruitment of TNF receptor-associated factors (TRAFs) followed by activation of transcription factors including AP-1 and NF-*κ*B.

Our analysis found that we were able to predict TNF*α *output with high accuracy, being able to predict 75% of its variance, second only to G-CSF. With this PLS model, we required only 6 latent variables to achieve this level of accuracy. Although the PCR model with time-averaged metrics required the use of 13 principal components, this model was able to achieve the highest predictive accuracy with an *R*^2 ^value of 0.84. Identification of key regulators of TNF*α *response with PLS agreed with those found in the literature [15,16]. Specifically, the PLS model identified the activity level of NF-*κ*B at 10 and 30 minutes and the total activity of JNK (considered here to be represented by the mean, maximum and AUC metrics extracted from the activation time course) as being the key predictive factors in this response, with both being in the top 10% of VIP variables (see Table 3).

As a final example, model predictions for the chemokine RANTES ('Regulated upon Activation, Normal T-cell Expressed and Secreted' or CCL5) were analyzed. RANTES is chemotactic for T cells, eosinophils and basophils, and is needed for the maintenance of allergic inflammation [17]. Model predictions for RANTES had *R*^2 ^values of 0.65 with 2 LVs/PCs when using time-dependent signaling metrics (Table 2). Furthermore, model predictions indicated that metrics associated with the activity of JNK and ERK1/2 to be in the top 10% of explanatory variables, which is supported in the literature [18,19].

**Figure 4:**
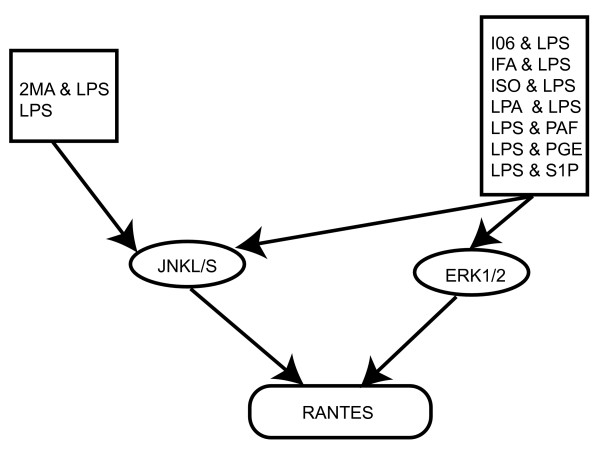
**Signaling network topology for RANTES based on the top ten signaling metrics**. The kinases JNK and ERK1/2 were found to play an important role in regulating RANTES from the PLS analysis. Legends shown in the top row were identified directly from the data (i.e. not a model output) as the top activators of either JNK or ERK1/2. Also see Table 3.

Introduced earlier, Figure 2 shows the squared weighted VIP profile for RANTES. This information is further summarized in a basic network diagram that highlights the most important input and signaling variables as determined through the model (Figure 4). Here the top 10% time-related, activation state properties of JNK and ERK1/2 are shown in the middle row. Also shown in the network for both JNK and ERK1/2 are the top 5 most significant input stimuli (i.e. those stimuli that caused the greatest increase in JNK or ERK1/2 phosphorylation state).

## Discussion

While significant work lies ahead, continued efforts in systems approaches to understanding cellular function hold considerable promise. Key to this success is the development and application of computational methods capable of synthesizing predictive models from large and complex data sets. Significant progress is being made in this area, with the application of multivariate approaches such as the PLS method described here being just one of many. Part of the usefulness of this approach for such high-level or "top-down" modeling, lies in its ability to decrease model complexity by reducing the number of problem dimensions to the smallest, most informative set. Greater reduction in model dimensions and the ability to rank and extract the most important model variables through VIP scores present tangible advantages over PCR.

**Figure 5:**
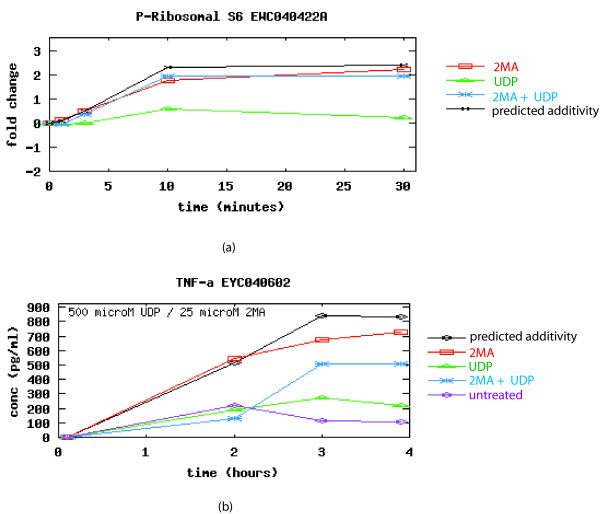
**Protein phosphorylation and cytokine concentration time courses after applying 2MA, UDP, or both**. (a) Protein phosphorylation of P-Ribosomal S6 was measured at 0, 1, 3, 10 and 30 minutes. (b) TNF*α *was measured at 0, 2, 3, and 4 hours. Longer time courses with greater sampling may be required to generate reliable results as many curves appeared to have been just initiated (source: [20] – see text for more details).

It should be noted that these high-level systems models determine the important modulators of system response by fitting model variables across all experimental conditions. As a result, some key proteins may be missed under certain circumstances. For example, protein activities that are primary drivers of a specific output in only a small number of experimental conditions may not be characterized as "significant." This enforces the concept that these models must be continually refined so as to address condition-specific details and be used to supplement more thorough investigations of pathways of interest. As described earlier, a major aim of this work was to determine if the addition of temporal information/metrics into the model would help to improve predictions. This was especially of interest as this data consisted of relatively few (4) time points. Results indicate that the use of this limited temporal information provided generally poor results, improving predictions in only 2 of 7 cases when compared to time-averaged data. We found that for many of the time courses, the response curves appear to have been just initiated, show relatively little dynamics, and/or are far from returning to post-stimulus levels. An example experimental time course is shown in Figure 5.

**Figure 6:**
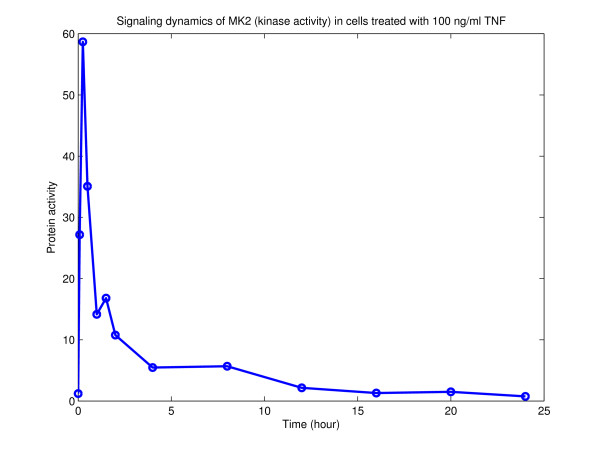
**Measured activity of protein kinase MK2**. Example of a 13 time point activity curve of the signaling dynamics of MK2 in HT-29 cells treated with 100 ng/ml TNF (Data from [4]).

Previous work using PLS with nearly identical data types has been shown to be highly effective, with model predictions having 90% correlation with measured outputs [4]. However, in this instance, protein activity curves initiated by pro-death and pro-survival cytokines were sampled at 13 time points between 0 to 24 hours, providing a more thorough picture of the temporal dynamics of protein signaling than the AfCS data (e.g. see Figure 6). Is the greater number of time points responsible for the improved performance? We performed an equivalent analysis of the data in [4] and focused on the effect of iteratively removing sampled timepoints, in various numbers and combinations, from the protein activity/signaling curves (data not shown). We found that, for this data, the initial 3–4 points sampled at the early stages of protein signaling, along with their associated metrics, were sufficient to give reliable predictions with over 80% accuracy. Thus it would appear that, at least in this case, a small number of properly placed samples can be sufficient to provide basic, but reliable characterization of signaling dynamics.

Together, these results emphasize that similar, systems-level studies should carefully consider the minimum number of time points that must be sampled in order to appropriately monitor system dynamics. Sampling need not be uniform and/or the same for all variables, but should rather be chosen so as to capture the desired dynamic properties of each variable (e.g. an initial rapid rise in protein phosphorylation state). Such experimental design decisions would be expected to significantly improve prediction accuracy as well as model interpretation, even with sparsely sampled protein signaling curves.

For systems biology approaches to succeed, modeling and experimental approaches must be highly integrated, and for models to provide worthwhile information, experiments must be designed so as to maximize the capabilities of the computational methods. The RAW 264.7 single and double-ligand AfCS data used in this study presents two issues of note in this regard. One issue is the use of completely different cell cultures for the collection of signaling protein phosphorylation state and cytokine secretion data. While the effects of this can presumably be monitored with a sufficient number of samples, this may raise questions in downstream analysis. Perhaps of even greater concern is the finding that in approximately 80% of experiments, the concentration of an applied stimulant used for the induction of protein phosphorylation was not the same concentration of stimulant used to induce a cytokine response. Thus stimuli conditions across experiments cannot be matched, leading to a significant reduction in the total amount of useable data. One would also expect that the lack of matching stimuli concentrations would generate significant errors in downstream predictions. As evidence of this, when we ignore differences in input concentrations and the complete set of data is used to develop a model for the prediction of RANTES output, we find that the quality of predictions is quite poor with an *R*^2 ^value of 0.31. With time-dependent signaling metrics, using the appropriately matched data sets only however, gives us the ~0.65 value described in this paper. The set of high-scoring VIP variables would also be expected to be biased, identifying incorrect variables as being key modulators of system behavior. While the original design of experiments may not have had computational analyses of data in mind, such computation-experiment design seriously impacts the broader utility of such data. Future efforts in this area will benefit greatly from building upon lessons learned in these still relatively new forays into systems biology.

## Materials and methods

### Data

RAW 264.7 single and double-ligand screen data were obtained from the AfCS Data Center. After applying single or double-ligand stimuli, phosphorylation changes of 21 signaling proteins were measured at 1, 3, 10 and 30 minutes, intracellular cAMP concentrations were measured at 20, 40, 90, 300 and 1200 seconds, and extracellular cytokine concentrations were measured at 2, 3 and 4 hours after initial stimulation [20].

### Data pre-processing

Since the concentrations for many ligands were different between the protein phosphorylation and cytokine secretion experiments, the AfCS data were first filtered to select matched ligand-stimulus conditions. From a total of 253 stimulant conditions, including both single and double-ligand stimuli, this filtering resulted in 55 conditions where the input stimuli concentration was identical for both the phosphorylation and cytokine experiments. These 55 conditions were then used in subsequent analyses [see Additional file 2 for details].

Protein phosphorylation data measuring a total of 55 stimulant conditions (including both single and double-ligand stimulus) was used to construct a predictor matrix (independent block). Note that cAMP data was not used in this analysis as it was only measured under a highly limited set of stimulant conditions (35 out of the original 253 stimulating conditions). For phosphorylation data, a fold change over baseline was first calculated [20] and the natural logarithm was subsequently taken. Since most measurements had at least three replicates, a four time-point time course defined as the mean signal at each time point was obtained for each protein. To extract as much information on the temporal dynamics as possible, we defined 11 time-dependent signaling metrics (Table 1) from each protein's time course, resulting in a 231-dimensional signaling space. Each time-dependent metric (for example, the log-transformed fold change of STAT5 at 1 min) was then divided by its standard deviation calculated across all stimulant conditions so as to maintain the relative variation in the data. For comparison purposes, a time-averaged predictor matrix was constructed for each protein by averaging across both replicates and time points. Thus for each protein, a unit-variance scaled single measurement was obtained under each ligand stimulus condition.

Seven cytokines (G-CSF, IL-1*α*, IL-6, IL-10, MIP-1*α*, RANTES and TNF*α*) with an average signal-to-noise ratio higher than five were chosen for further analysis (as in [7]). A predicted vector (dependent block) was constructed for each cytokine as follows. For each cytokine, 1 was added to both the baseline and the measured concentrations, and then a fold change over the baseline was calculated prior to the natural logarithm transformation. Finally, a unit-variance scaled three time-point time course was obtained for each cytokine.

We also looked at whether the greater number of time points/metrics was responsible for the improved performance shown in [4], a work which also used PLS to model nearly identical data types. To do this, we performed an equivalent analysis of the data in [4] and focused on the effect of removing sampled timepoints, in various numbers and combinations, from the original protein activity/signaling curves – e.g., generate a PLS model based on only the first four time points and associated metrics. Note that, during the generation of peak metrics (AUC, activation slope and decay rate of each peak), we only consider the two most significant peaks for a given time course. As in [4], no log transformations of the data were performed.

### Partial least squares regression

PCR models were constructed in Matlab (Mathworks Inc.) with PLS models being constructed via the SIMPLS algorithm [21] using the PLS toolbox (Eigenvector Research).

Ten-fold cross-validation was performed to select the optimum number of latent variables used in the regression models. For each cytokine output response, the dataset was split into ten equally sized folds/subsets. A regression model was then constructed using all but one of the subsets (calibration-step) using up to 100 model components. This model was then used to estimate the samples in the left-out fold [22]. After iteration through all ten subsets, the RMSEs of both the calibration and the cross-validation were then plotted as functions of the number of LVs used. Normally, the calibrated RMSE decreases monotonically, but the RMSECV should be minimized with a certain number of latent variables, from which the optimal number of LVs may be determined for each cytokine. The squared Pearson correlation coefficients between the predicted and measured cytokine values, *R*^2^, were also computed for each cytokine to assess the quality of the prediction. This approach was mirrored in the PCR analysis where cross-validation was also performed to determine the ideal number of PCs and the *R*^2 ^value for each cytokine prediction.

### Dimension reduction and model averaging

When assigning significance to each explanatory predictor (e.g., AUC for STAT3) in the model, the VIP score of each predictor is usually computed from the PLS regression model. These VIP scores estimate the importance of each predictor variable used in the PLS model and are often used to select those predictors that are most influential in a given output response [23]. If a predictor has a small VIP score, it is considered to be a prime candidate for removal from the final regression model. By removing less important variables from the model and keeping those that are of predictive value, we can obtain sufficient prediction accuracy while simultaneously minimizing the number of variables within the model that need to be measured.

To reduce the prediction bias and the likelihood of overfitting, we again used cross-validation to obtain test samples that were different from training samples. By doing this, a more realistic estimate of the prediction error can be obtained. In this work, *M *= 10 PLS models were constructed through a 10-fold cross-validation procedure for each cytokine. Each generated model (derived from a particular set of test data) has its own level of prediction accuracy. In practice, however, often no single candidate model is obviously superior to the others. More difficult to reconcile is the case where VIP score profiles differ markedly across all candidates' models, making it inappropriate to select vital predictors based on a single candidate model. As a result, in this work we computed a weighted VIP score to select important signaling metrics based on all ten PLS models using a simple and efficient model averaging approach for each cytokine (described below). By performing model averaging and then selecting only the most influential variables, we are able to create meaningful "minimal" models that are able to predict the cytokine output response with good accuracy.

Assuming all models have normally distributed residual errors with a constant variance, an adjusted small sample Akaike's Information Criterion (AIC) score was determined for each model from least squares regression statistics [24,25]:

$$AIC_{m}=N\log{\overset{\wedge }{\sigma }}_{m}^{2}+2K+\frac{2K(K+1)}{N-K-1}(m=1,\cdots ,M)$$

where *K *is the number of estimated regression parameters, *N *is the number of stimulant conditions and ${\overset{\wedge }{\sigma }}_{m}^{2}=\frac{\sum_{i=1}^{N}{\overset{\wedge }{\varepsilon }}_{m,i}^{2}}{N}$ and ${\overset{\wedge }{\varepsilon }}_{m,i}^{2}(i=1,\cdots ,N)$ are the estimated residuals for the *m*th candidate model. For a given cytokine, since the same number of LVs was assumed for all candidate models and all ten subsets have the same size, *K *is the same for all models. Hence, the adjusted small sample AIC is proportional to:

$$AIC_{m}\propto N\log{\hat{\sigma }}_{m}^{2}(m=1,\cdots ,M)$$

To allow a quick comparison and ranking of candidate models, the difference between AIC scores was computed over all candidate models and for each cytokine as

$$\Delta_{m}=AIC_{m}-\underset{i⩽m⩽M}{\min}AIC_{m}(m=1,\cdots ,M)$$

To better interpret the relative likelihood of each candidate model, the Akaike weight for each model is determined by [26,27]:

$$\omega_{m}=\frac{e^{-\Delta_{m}/2}}{\sum_{m=1}^{M}e^{-\Delta_{m}/2}}(m=1,\cdots ,M)$$

where the Akaike weight *ω*_*m *_represents the evidence in favor of the *m*th candidate model being the best Kullback-Leibler (K-L) model. Note that this assumes that one of the *M *models is also the K-L best model.

For a given PLS regression model, the VIP score for the *k*th signaling metric is computed as:

$$VIP_{m,k}=\sqrt{\frac{P\sum_{i=1}^{R}\frac{w_{m,ik}^{2}SS_{m,i}}{||w_{m,i}|{|}^{2}}}{\sum_{i=1}^{R}SS_{m,i}}}$$

where *m *= 1, ⋯, *M*; *k *= 1, ⋯, *P*, *P *is the number of total signaling metrics, *w*_*m, ik *_is the weight of the *k*th metric for the *i*th latent variable in the *m*th model, *R *is the number of LVs, and *SS*_*m, i *_is the sum of squares explained by the *i*th LV in the *m*th model [28]. The weighted VIP score is then determined by:

$$wVIP_{k}=\sqrt{\frac{\sum_{m=1}^{M}\omega_{m}VIP_{m,k}^{2}}{\sum_{m=1}^{M}\omega_{m}}}(k=1,\cdots ,P)$$

Since the average of the squared weighted VIP scores equals one, important metrics were defined as any signaling metric with a weighted VIP score greater than 1.

## Authors' contributions

YW, GLJ, and SMG conceived the project, YW and SMG performed the research, YW, GLJ and SMG analyzed the results. All authors read and approved the final manuscript.

## Supplementary Material

Additional file 1 **Segment diagrams of the squared weighted VIP profile for G-CSF, IL-1*α*, IL-6, IL-10, MIP-1*α *and TNF*α***. For each cytokine, ten PLS models were generated through a 10-fold cross validation and then a weighted VIP score was computed as described in Materials and Methods to select important signaling metrics. A segment plot was produced for each protein, with the radial length of each segment indicating the magnitude of the squared weighted VIP score for individual metrics. VIP scores greater than 1 (dashed circle) are classified as significant metrics for each cytokine. Click here for file

Additional file 2 **Experimental conditions used in model creation**. These conditions were identical for both protein phosphorylation state and cytokine output response measurements. Abbreviations: 2MA – 2-Methyl-thio-ATP; IFA – Interferon-alpha; IL-6 – Interleukin-6; ISO – Isoproterenol; LPA – Lysophosphatidic acid; LPS – Lipopolysaccharide; PAF – Platelet Activating Factor; PGE – Prostaglandin E2; S1P – Sphingosine-1-phosphate; UDP – Uridine 5'-diphosphate. Click here for file
