# Supplementary material for: Data-driven modeling of cellular stimulation, signaling and output response in RAW 264.7 cells
Source: J Mol Signal. 2008 May 22;3:11. doi: 10.1186/1750-2187-3-11 (PMC2441624; doi:10.1186/1750-2187-3-11)

Supplemental Figure 1: Segment diagrams of the squared weighted VIP profile for G-CSF, IL-1α, IL-6, IL-10, MIP-1α and TNFα. For each cytokine, ten PLS models were generated through a 10-fold cross validation and then a weighted VIP score was computed as described in Materials and Methods to select important signaling metrics. A segment plot was produced for each protein, with the radial length of each segment indicating the magnitude of the squared weighted VIP score for individual metrics. VIP scores greater than 1 (dashed circle) are classified as significant metrics for each cytokine.

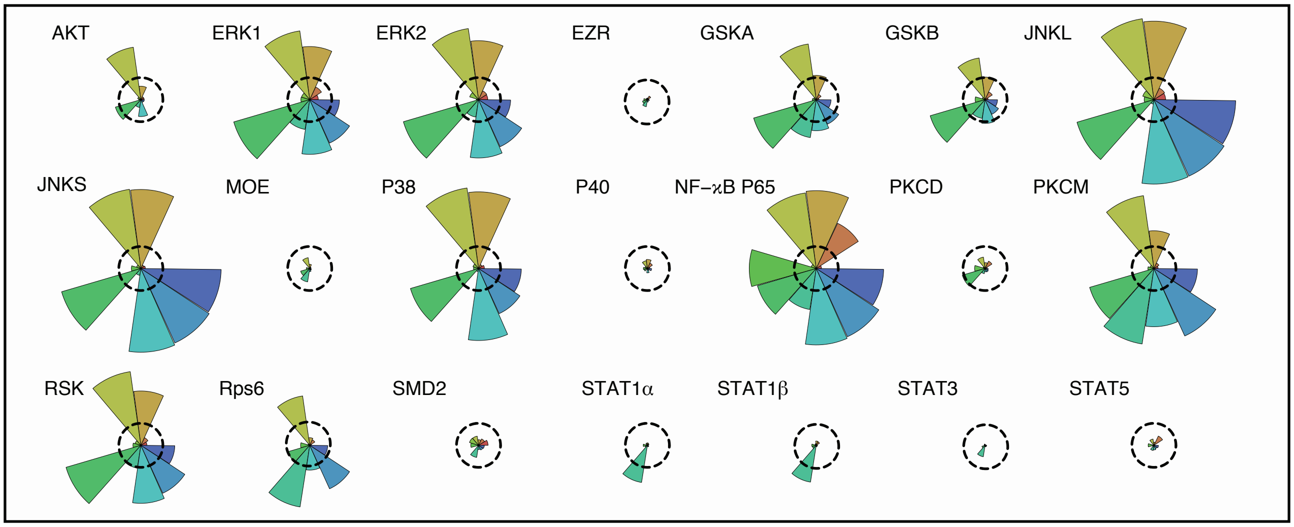

(a) G-CSF

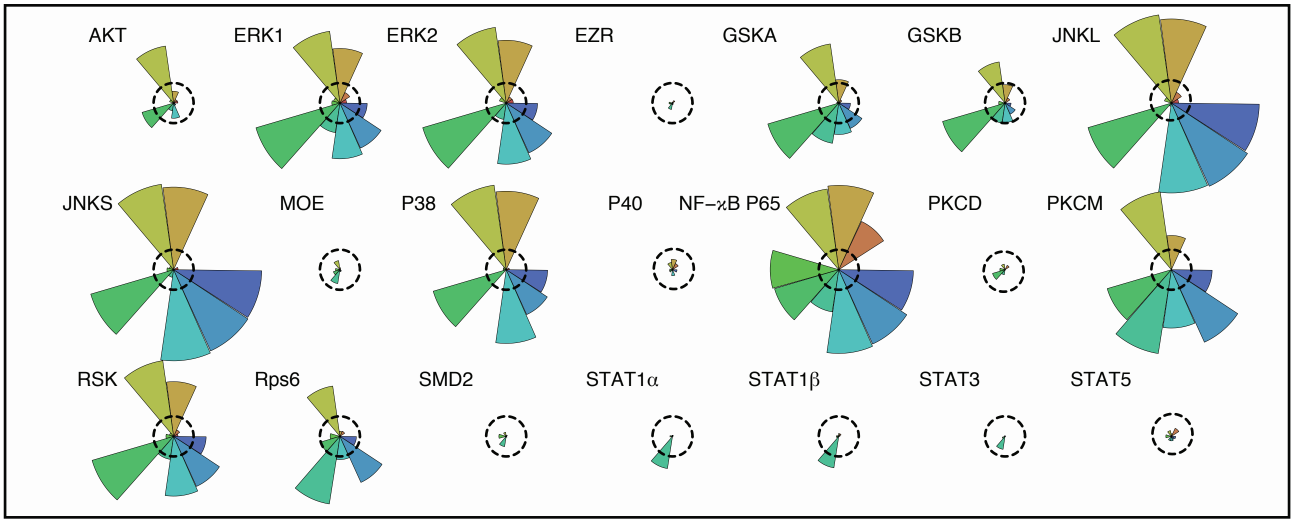

(b) IL-1α

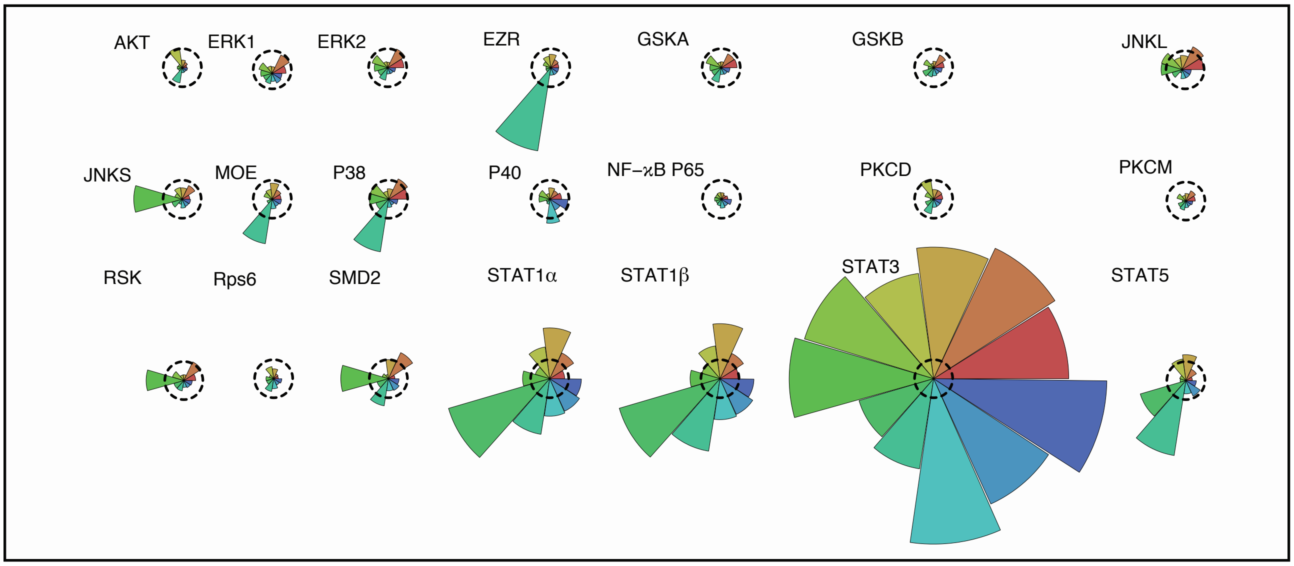

(c) IL-6

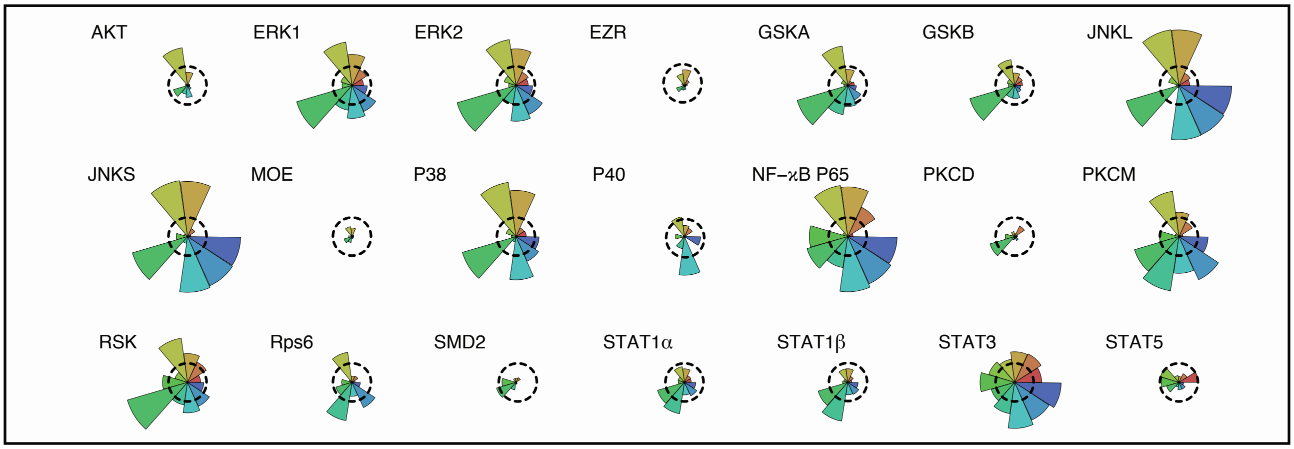

(d) IL-10

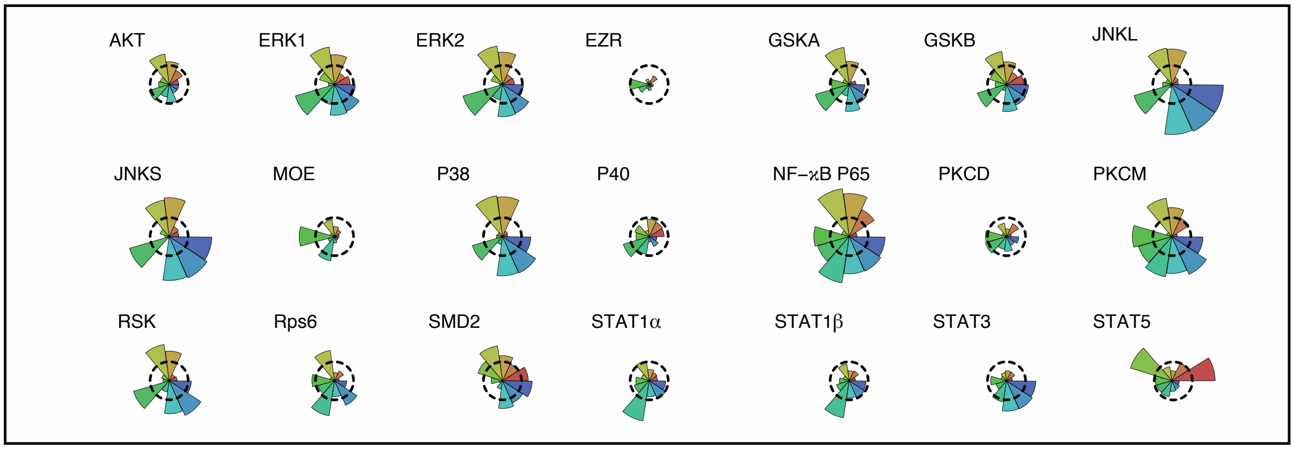

(e) MIP-1α

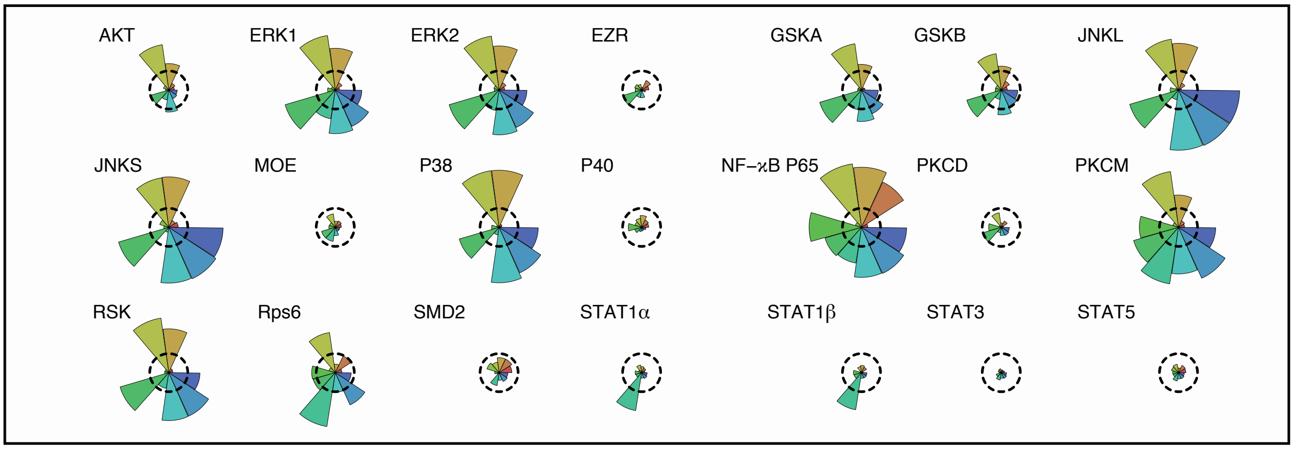

(f) TNFα

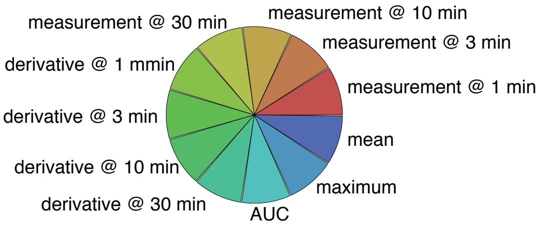

Supplement: Additional file 1 — Segment diagrams of the squared weighted VIP profile for G-CSF, IL-1α, IL-6, IL-10, MIP-1α and TNFα. For each cytokine, ten PLS models were generated through a 10-fold cross validation and then a weighted VIP score was computed as described in Materials and Methods to select important signaling metrics. A segment plot was produced for each protein, with the radial length of each segment indicating the magnitude of the squared weighted VIP score for individual metrics. VIP scores greater than 1 (dashed circle) are classified as significant metrics for each cytokine. [file 1750-2187-3-11-S1.pdf]
