# Supplementary material for: Data-driven modeling of cellular stimulation, signaling and output response in RAW 264.7 cells
Source: J Mol Signal. 2008 May 22;3:11. doi: 10.1186/1750-2187-3-11 (PMC2441624; doi:10.1186/1750-2187-3-11)
Supplement: Additional file 2 — Experimental conditions used in model creation. These conditions were identical for both protein phosphorylation state and cytokine output response measurements. Abbreviations: 2MA – 2-Methyl-thio-ATP; IFA – Interferon-alpha; IL-6 – Interleukin-6; ISO – Isoproterenol; LPA – Lysophosphatidic acid; LPS – Lipopolysaccharide; PAF – Platelet Activating Factor; PGE – Prostaglandin E2; S1P – Sphingosine-1-phosphate; UDP – Uridine 5'-diphosphate. [file 1750-2187-3-11-S2.pdf]

Supplemental Table 1: Experimental conditions used in model creation

	Ligand 1	Concentration	Ligand 2	Concentration
	2MA	500 µM		
	IL-6	300 pM		
	IFA	1 nM		
	ISO	50 nM		
	LPA	2.5 µM		
	LPS	100 ng/mL		
	PAF	100 nM		
	PGE	10 µM		
	S1P	1 µM		
	UDP	25 µM		
	2MA	500 µM	IL-6	300 pM
	2MA	500 µM	IFA	1 nM
	2MA	500 µM	ISO	50 nM
	2MA	500 µM	LPA	2.5 µM
	2MA	500 µM	LPS	100 ng/mL
	2MA	500 µM	PAF	100 nM
	2MA	500 µM	PGE	10 µM
	2MA	500 µM	S1P	1 µM
	2MA	500 µM	UDP	25 µM
	IL-6	300 pM	IFA	1 nM
	IL-6	300 pM	ISO	50 nM
	IL-6	300 pM	LPA	2.5 µM
	IL-6	300 pM	LPS	100 ng/mL
	IL-6	300 pM	PAF	100 nM
	IL-6	300 pM	PGE	10 µM
	IL-6	300 pM	S1P	1 µM
	IL-6	300 pM	UDP	25 µM
	IFA	1 nM	ISO	50 nM
	IFA	1 nM	LPA	2.5 µM
	IFA	1 nM	LPS	100 ng/mL
	IFA	1 nM	PAF	100 nM
	IFA	1 nM	PGE	10 µM
	IFA	1 nM	S1P	1 µM
	IFA	1 nM	UDP	25 µM
	ISO	50 nM	LPA	2.5 µM
	ISO	50 nM	LPS	100 ng/mL
	ISO	50 nM	PAF	100 nM
	ISO	50 nM	PGE	10 µM
	ISO	50 nM	S1P	1 µM
	ISO	50 nM	UDP	25 µM
	LPA	2.5 µM	LPS	100 ng/mL
	LPA	2.5 µM	PAF	100 nM
	LPA	2.5 µM	PGE	10 µM
	LPA	2.5 µM	S1P	1 µM
	LPA	2.5 µM	UDP	25 µM
	LPS	100 ng/mL	PAF	100 nM
	LPS	100 ng/mL	PGE	10 µM
	LPS	100 ng/mL	S1P	1 µM
	LPS	100 ng/mL	UDP	25 µM
	PAF	100 nM	PGE	10 µM
	PAF	100 nM	S1P	1 µM
	PAF	100 nM	UDP	25 µM
	PGE	10 µM	S1P	1 µM
	PGE	10 µM	UDP	25 µM
	S1P	1 µM	UDP	25 µM

These conditions were identical for both protein phosphorylation state and cytokine output response measurements. Abbreviations: 2MA – 2-Methyl-thio-ATP; IFA – Interferon-alpha; IL-6 – Interleukin-6; ISO – Isoproterenol; LPA – Lysophosphatidic

acid; LPS – Lipopolysaccharide; PAF – Platelet Activating Factor; PGE – Prostaglandin E2; S1P - Sphingosine-1-phosphate; UDP – Uridine 5'-diphosphate.
